# Effects of Palatable Food Versus Thin Figure Conflicts on Responses of Young Dieting Women

**DOI:** 10.3389/fpsyg.2019.01025

**Published:** 2019-05-22

**Authors:** Shuaiyu Chen, Todd Jackson, Debo Dong, Qian Zhuang, Hong Chen

**Affiliations:** ^1^ Key Laboratory of Cognition and Personality, Ministry of Education, Southwest University, Chongqing, China; ^2^ School of Psychology, Southwest University, Chongqing, China; ^3^ Department of Psychology, University of Macau, Macau, China; ^4^ School of Life Science and Technology, University of Electronic Science and Technology of China, Chengdu, China

**Keywords:** dieting, appetitive-driven motivation, figure-driven motivation, food vs. figure conflict, automatic processing bias

## Abstract

Many young women use dieting to achieve a thinner figure yet most tend to fail as a result of heightened responsiveness to palatable food environments and increases in hedonic cravings. In this preliminary study, we developed a novel palatable food vs. thin figure conflict task to assess conflicting motives associated with eating among young women. Forty young dieting women [mean body mass index (BMI) = 22.98 kg/m^2^, *SD* = 3.81] completed a food vs. figure conflict task within a 2 (distractor image: food vs. figure) × 2 (word-image congruence: congruent vs. incongruent) within-subjects design. Results supported the view that this new task could effectively capture conflict costs. Dieting young women displayed stronger food conflicts than figure conflicts based on having longer response delays and higher error rates in the food conflict condition than the figure conflict condition. Although young women often proclaimed “dieting” to achieve or maintain a good figure, dieters appeared to exhibit stronger preferences for palatable food cues relative to thin figure cues. These results provide important information for understanding automatic processing biases toward palatable foods and underscore the need for research extensions in other cultural contexts to determine whether such biases are universal in nature.

## Introduction

In obesogenic environments, dieting has become so prevalent that it is a normative mode of eating, especially among young women (Andreyeva et al., 2010). Although many people use dieting as a weight loss or maintenance strategy, most are unsuccessful at dieting over extended timeframes (Mann et al., 2007). Furthermore, hedonic-craving urges increase for dieters exposed to food cues and often result in inappropriate intake behaviors, such as overeating and binge eating (Stice, 2001). Research has suggested that dieters usually regain much or all of their lost weight at a later time (Lowe et al., 2013). As a result, dieting has become a controversial strategy for losing weight (Lowe and Levine, 2005).

According to the goal conflict model of eating (Stroebe et al., 2013), dieters often fail because they are confronted with conflicting goals: appetitive enjoyment versus weight control. Activation of the appetitive enjoyment goal owing to heightened exposure to palatable food cues may inhibit access to mental representations of the weight control goal and consequently lead to dieting violations and unhealthy eating (Stroebe et al., 2013). On the other hand, previous studies have found dieters often have two specific motivations for weight control: improving health (i.e., physical fitness) and achieving a thinner figure (Chapman, 1999; Putterman and Linden, 2004). Some research indicates that younger female dieters more often endorse dieting for the purpose of having a thinner figure than physical fitness (Putterman and Linden, 2004; Chithambo, 2018). Consequently, young women who diet may experience a conflict between the appetitive enjoyment and having a thinner figure. Unfortunately, compared to cohorts who engage in health-focused dieting, those who engage in figure-focused dieting may be at a higher risk for losing control of eating because they are more prone to using drastic or harmful dieting strategies (Putterman and Linden, 2004; Haynos et al., 2015).

From the perspective of cognitive control, conflict arises when processing of goal-congruent information is disrupted by goal-incongruent distractors (Botvinick et al., 2001), such as salient food cues. To date, laboratory studies have yet to examine effects of conflict between appetitive enjoyment versus appearance goals on dieters. Drawing from the logic of conflicting information processing within Stroop color naming tasks, we created a picture-word interference paradigm designed to assess effects of food versus figure goal conflicts on responses of young dieting women (Damian and Bowers, 2003). Specifically, palatable food words and thin figure words were superimposed over both palatable food images and thin figure images. For each trial, participants were instructed to identify the relevant stimulus dimension (i.e., target word category) and ignore the irrelevant stimulus dimension (i.e., accompanying distractor context). In general, relative to “congruent” picture-word category presentations, “incongruent” picture-word presentations should be associated with longer reaction times (RT) and reduced accuracy rates that reflect conflict effects. This preliminary study was based on the premise that dieters have more frequent food cravings and stronger disinhibited eating than non-dieters do (Rideout and Barr, 2009; Massey and Hill, 2012). As such, young dieting women should display more conflict (i.e., slower RT, lower accuracy rates) when food images rather than figure images were used as distractors.

## Materials and Methods

### Participants

Participants were 40 right-handed young women who reported dieting at present. Based on previous research (Lowe et al., 2006; Buckland et al., 2014), participants who were “currently dieting to maintain or lose weight” were recruited initially from Southwest University, Chongqing *via* an online participant recruitment platform (Sojump). Prospective volunteers were queried for the following exclusion criteria: current pregnancy, taking contraceptive drugs, current or past psychiatric illness, an eating disorder or history of disordered eating. Three participants were excluded due to reports of high-intensity acute exercise or sleeplessness. The final sample (*N* = 40) had an average body mass index (BMI: kg/m^2^) of 22.98 (*SD* = 3.81). Table 1 summarizes characteristics of the sample.

**Table 1:** Demographic characteristics of sample.

Variable	Range	*M* (*SD*)
Age (years)	18–24	19.90 (1.35)
BMI	17.7–30.7	22.98 (3.81)
Fast time (h)	2–22	5.95 (5.16)
Hunger level	0–80	41.40 (24.58)

*BMI, body mass index*.

This study was approved by the Human Research Ethic Committee in the School of Psychology, Southwest University. All participants completed an informed consent form that included a general overview of the study and its requirements, the right to withdraw at any point without penalty, and statements about compliance with data protection guidelines in accordance with the Declaration of Helsinki.

### Materials

#### Word Stimuli

Original thin figure words (*n* = 18) were based on other published research (Chen et al., 2006; Weng et al., 2012). An independent sample of 25 young women was recruited to assess the eight words with which they were most familiar that best described women’s ideal figure. Of these, the four most frequently endorsed figure words (i.e., miaotiao, xianshou, xiuchang, and xiaomanyao, Chinese characters) were used in the research. Palatable food words were also drawn from published research (Weng et al., 2012). Subsequently, 80 young women rated the palatability and familiarity of food words and their perception of the extent to which the associated food would cause weight gain. Based on these ratings, four highly palatable, familiar, high weight gain food words (i.e., cake, chips, chocolate, and chicken steak) were selected. Figure and food word categories were also matched for number of strokes in Chinese characters.

#### Figure and Food Images

Eight thin figure images that featured the body but excluded the head were adapted from previous published research based on the assessment of attractiveness (Gao et al., 2013). Eight food images were selected from a Chinese food picture database based on the assessment of palatability as above and each food having two corresponding images. Images of food and figure were matched for size, resolution, brightness, and background.

#### Demographics

Participants completed demographic items related to age, height, and weight.

### Procedure

Prior to their laboratory sessions, participants were instructed to refrain from eating or drinking any liquids except water for at least 2 h before their appointments. Upon arrival, participants read and signed the informed consent. Next, they reported their fasting time and hunger level from 0 (not hungry at all) to 100 (very hungry). Subsequently, participants performed the food vs. figure conflict task described below. Following completion of the task, participants were debriefed about the main research purpose, paid 30 yuan, and thanked for their participation.

### Food Versus Figure Word Image Conflict Task

As noted above, palatable food words and thin figure words written in outer glowing font were overlaid on both thin figure and palatable food pictures. Target words were either incongruent or congruent with distracting pictures, resulting in four types of stimulus trials: food words – food images (food-congruent trials), thin figure words – thin figure images (figure-congruent trials), figure word – food images (food-incongruent trials), and food words – figure images (figure-incongruent trials). Stimulus pairs were presented for 1,000 ms. Participants were instructed to categorize words as “food” or “figure” by pressing corresponding response buttons while ignoring the accompanying picture distracters. The assignment of the response buttons was counterbalanced across participants. Stimulus trials were presented in a pseudo-random order with the constraint that there was no repetition on two consecutive trials (Mayr et al., 2003). A fixation cross was presented before each stimulus pair, with a varying time of 1,500–2,500 ms. Following past research (Yang et al., 2016; Fan et al., 2018; Kar et al., 2018), the task comprised three blocks of 80 trials each resulting in a total of 240 trials with 60 trials per condition. All women received a practice task of 20 trials with different stimuli than those used in the main research task.

Based on previous studies (Etkin and Schatzberg, 2011; Tillman and Wiens, 2011), the main conflict index was calculated by subtracting the mean RT of correct congruent trials for food and figure distracting conditions, respectively, from the mean RT of corresponding correct incongruent trials. Calculations resulted in food-conflict and figure-conflict indexes.

## Results

**Figure 1:**
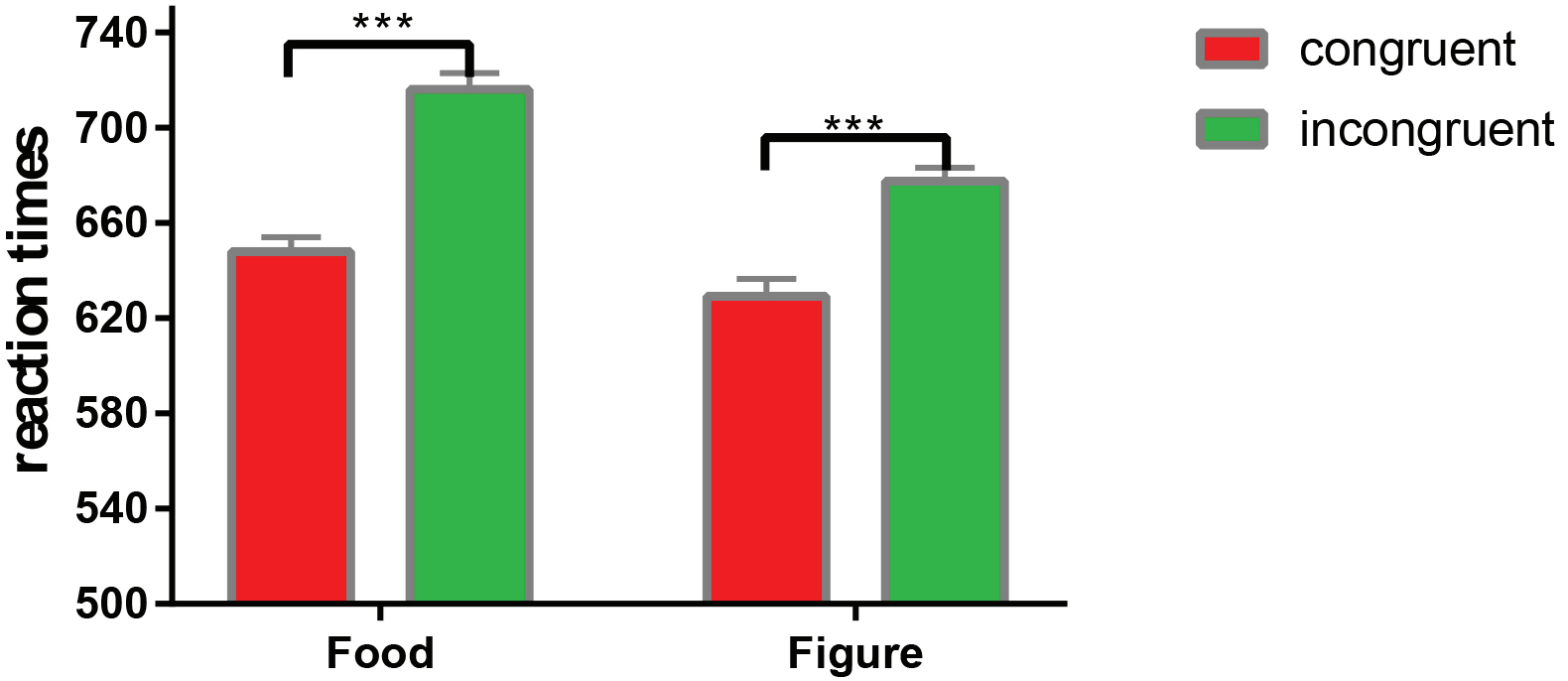
The interaction effect of reaction times on the congruent and incongruent trials. ***p < 0.001.

To test the premise that incongruent word-image trials would generate slow reaction times than would congruent word-image trials, a 2 (word-image congruence: congruent vs. incongruent) × 2 (distractor image: food vs. figure) repeated measures ANCOVA analysis was performed on RT; hunger, fasting time, and BMI were covariates in the analysis. There were significant main effects of congruence, *F*_(1, 36)_ = 545.31, *p* = 0.001, *η*^2^ = 0.94, distractor context, *F*_(1, 36)_ = 144.59, *p* = 0.001, *η*^2^ = 0.8, and their interaction, *F*_(1, 36)_ = 8.32, *p* = 0.007, *η*^2^ = 0.19. Simple effect analyses revealed that the mean RT of incongruent trials (*M* = 716.12, *SD* = 43.09) was significantly slower than the mean RT of congruent trials (*M* = 648.01, *SD* = 37.79) in the food-distractor context, *F*_(1, 36)_ = 355.83, *p* = 0.001, *η*^2^ = 0.91. The mean RT of incongruent trials (*M* = 677.62, *SD* = 34.24) was also significantly slower than the mean RT of congruent trials (*M* = 629.24, *SD* = 46.58) in the figure-distractor context though the effect size was smaller, *F*_(1, 36)_ = 102.76, *p* = 0.001, *η*^2^ = 0.74 (Figure 1).

**Figure 2:**
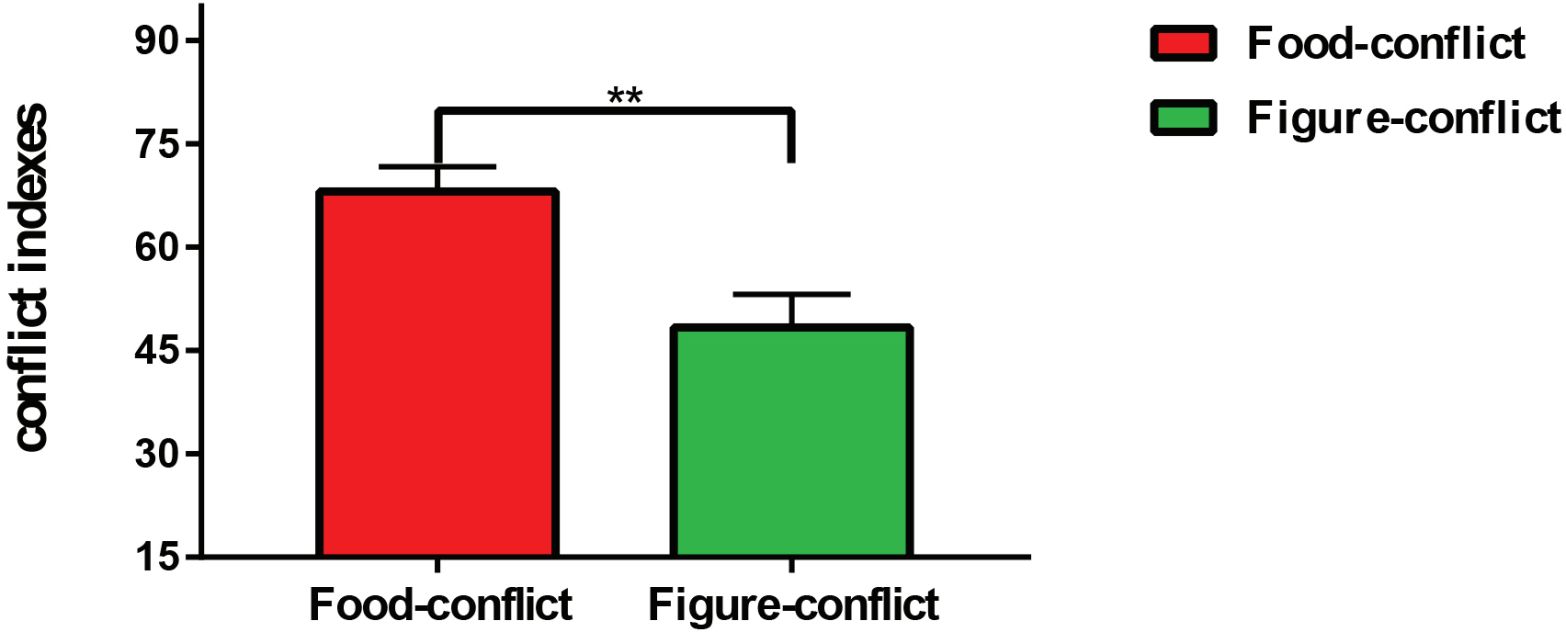
The difference between food-conflict and figure-conflict. **p < 0.01.

Subsequently, we directly compared difference in conflict indexes using the above covariates. Results showed that the food-conflict index (*M* = 68.11, *SD* = 22.25) was significantly greater than the figure-conflict index (*M* = 48.39, *SD* = 29.59), *F*_(1, 36)_ = 8.32, *p* = 0.007, *η*^2^ = 0.19 (Figure 2).

**Figure 3:**
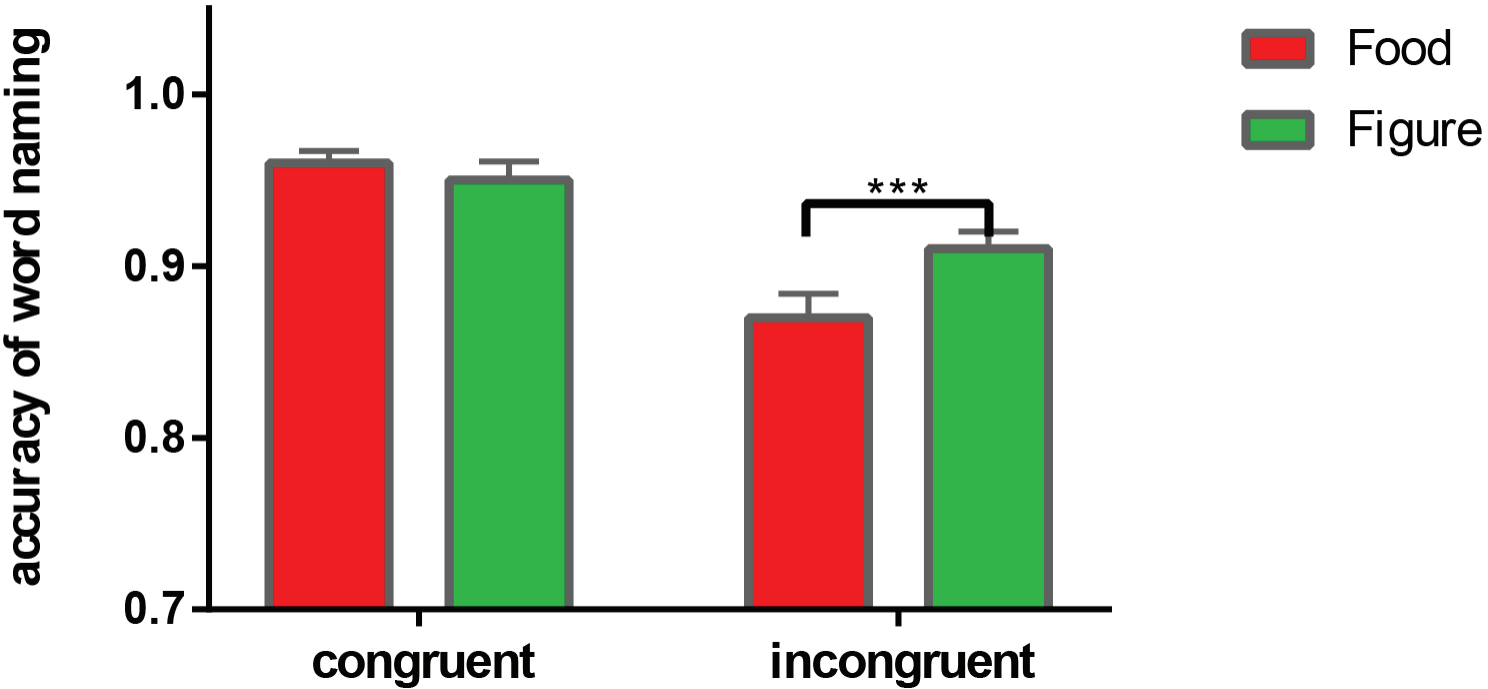
The interaction effect of accuracy rate on the congruent and incongruent trials. ***p < 0.001.

A 2 (word-image congruence: congruent vs. incongruent) × 2 (distractor image: food vs. figure) repeated measures ANCOVA was also performed on accuracy rates using the above covariates. Significant effects were observed for congruence, *F*_(1, 36)_ = 117.56, *p* = 0.001, *η*^2^ = 0.77, distractor context, *F*_(1, 36)_ = 8.66, *p* = 0.006, *η*^2^ = 0.19, and their interaction, *F*_(1, 36)_ = 12.33, *p* = 0.001, *η*^2^ = 0.26. Simple effects analyses indicated the accuracy rate was significantly lower for food-incongruent trials (*M* = 0.87, *SD* = 0.08) than figure-incongruent trials (*M* = 0.91, *SD* = 0.06), *F*_(1, 36)_ = 26.66, *p* = 0.001, *η*^2^ = 0.43, while accuracy rates for food-congruent trials (*M* = 0.96, *SD* = 0.04) versus figure-congruent trials (*M* = 0.95, *SD* = 0.06) did not differ, *F*_(1, 36)_ = 1.57, *p* = 0.218, *η*^2^ = 0.04 (Figure 3).

## Discussion

According to the conflict monitoring and cognitive control model, incompatible information processing goals slow reaction times and hamper response accuracy, behavioral performance effects that are called conflict costs (Botvinick et al., 2001). Longer RT and lower accuracy rates for incongruent word-image trials than congruent trials confirmed that the paradigm developed in this study was suitable for measuring immediate responses to food vs. figure conflicts in line with conclusions of conceptually similar research (Damian and Bowers, 2003; Tillman and Wiens, 2011).

Notwithstanding young dieting women often attempt to lose weight to achieve or maintain a thinner figure (Putterman and Linden, 2004; Verstuyf et al., 2012), findings revealed that young dieting women experienced stronger conflict effects in response to food images rather than figure images used as distractors. Specifically, participants had slower reaction times and made more errors when in food images appeared as distractors in identifying figure words than a complementary incongruent condition featuring figure distractors and food words. These results reflected a stronger automatic processing bias toward palatable food imagery relative to thin figure imagery. These findings align with the assertion that dieting increases responsiveness to palatable foods and heightens hedonic cravings (Stice, 2001; Massey and Hill, 2012) that increase risk for problematic eating patterns (e.g., binge eating) and dieting failures over time (Stice et al., 2005; Lowe, 2015). Previous studies have found dieters are more immediately responsive to the hedonic value of food and display increased hypervigilance toward high-calorie foods even at a preattentive level (Fadardi and Bazzaz, 2011; Luomala et al., 2018). The current findings suggested that dieters may display a discrepancy between dieting intent and behavior in the context of competing food versus figure stimuli. Recent studies suggest that self-proclaimed “dieting” might refer to a commitment to restrict food consumption rather than actually engaging in the weight loss behaviors (Lowe and Levine, 2005; McLaughlin et al., 2018).

Human eating behaviors are largely influenced by automatic processes rather than deliberation upon the consequences of actions (Neal et al., 2006). According to the dual-process model (Hofmann et al., 2008), appetitive-driven motivation is an impulsive “hot” pathway that is characterized by effortless, automatic impulse tendencies in responding to palatable foods without awareness while thin figure-driven motivation reflects a “cold” pathway that is characterized by slow, goal-directed processing that relies on cognitive resources and noetic decisions. Dieters in this research showed stronger immediate preferences for palatable food cues than thin figure cues, suggesting appetitive-driven motivation overrides thin figure-driven motivation upon exposure to competing cues (Stroebe et al., 2013).

Although preliminary, this study may be the first to investigate motivational conflicts between appetitive enjoyment goal and thin figure goal within a group that is confronted with these competing goals (i.e., young dieting women). Nonetheless, its main limitations must be noted as a basis for future extensions. First, while the sample under study was appropriate for the main research purposes, it is not clear whether results would generalize to non-dieters, younger girls, and older women. The inclusion of such groups in extensions can elucidate the issue of specificity versus generalizability of effects reported here. Second, given that shape concerns, appearance ideals, and maladaptive eating patterns of men differ from those of women, future studies should examine the applicability of modified food-figure tasks to boys and men. Finally, it is not clear whether other variables, such as trait food cravings and figure concern (Chen et al., 2006; Moreno et al., 2009) moderate conflict indexes assessed in this research.

## Ethics Statement

This study was carried out in accordance with the recommendations of the ethical guidelines of the American Psychological Association. The protocol was approved by the Ethics Committee of School of Psychology, Southwest University. All participants gave written informed consent in accordance with the Declaration of Helsinki.

## Author Contributions

SC, HC, and TJ designed the research. SC, DD, and QZ collected and analyzed the data. HC conducted literature searches and provided summaries of previous research studies. SC and TJ wrote the manuscript, and all authors contributed to and have approved the final manuscript.

### Conflict of Interest Statement

The authors declare that the research was conducted in the absence of any commercial or financial relationships that could be construed as a potential conflict of interest.
